# Childhood Drug and Non-Drug Poisoning in Nigeria: An Economic Appraisal

**DOI:** 10.5334/aogh.2544

**Published:** 2019-07-09

**Authors:** Ifunanya Ikhile, Ifeyinwa Chijioke-Nwauche, Orish Ebere Orisakwe

**Affiliations:** 1 Department of Clinical Pharmacy, Faculty of Pharmacy, University of Port Harcourt, NG; 2 Department of Experimental Pharmacology and Toxicology, Faculty of Pharmacy, University of Port Harcourt, NG

## Abstract

**Background:**

The dearth of information on the economic cost of childhood poisoning in sub-Saharan Africa necessitated this study.

**Objective:**

This study has investigated the prevalence of childhood drug and non-drug poisoning, treatment modalities and economic costs in Nigeria.

**Method:**

A retrospective study of childhood drug and non-drug poisoning cases from January 2007 to June 2014 in the University of Port Harcourt Teaching Hospital (UPTH), Port Harcourt, Nigeria was carried out. Medical records were analysed for demographic and aetiological characteristics of poisoned children (0–14 years of age), as well as fiscal impact of poisoning cases.

**Findings:**

Of the 100 poisoned patients, 46% were male and 54% female, with female/male ratio of 1.17:1. Most of the children were under five years of age. Paracetamol, amitriptyline, chlorpromazine, ferrous sulphate, kerosene, organophosphates, carbon monoxide, snake bite, alcohol and rodenticides were involved in the poisoning. The average cost of poison management per patient was about $168, which is high given the economic status of Nigeria.

**Conclusion:**

Childhood poisoning is still a significant cause of morbidity among children in Nigeria and accounts for an appreciable amount of health spending, therefore preventive strategies should be considered.

## Background

Poisoning is an important global health problem [1] and one of the most common childhood medical emergencies. It remains a leading public health concern due to its frequency, severity, potential for death and disability and resultant hospitalization costs [2]. The epidemiological properties of childhood intoxication such as the kind of agents involved, type of intoxication and population at risk may differ by country depending on the lifestyle habits, geographical localization and other factors [3]. In order to understand the problem, determine economic impact and establish preventive and standard treatment measures special epidemiological surveillance for each country is necessary. While various international studies have shown the extent of the problem and associated fiscal impact, there is a paucity of published literature on the subject in sub-Saharan Africa. This knowledge gap has informed the conduct of a retrospective study of hospital medical records to describe the epidemiological features, incidence and outcome of hospitalization in poisoned paediatric patients, identify principal agents of poisoning and assess economic implications by fiscal impact analysis in a bid to reduce morbidity, mortality and costs related to childhood intoxication [3,4].

This paper explores the extent to which childhood poisoning has evolved over the years in Port Harcourt, Nigeria. It attempts to describe the pattern, socio demographic characteristics, as well as fiscal impact of drug and non-drug poisoning among children in Nigeria.

## Methodology

This is a hospital-based retrospective patient record study carried out in the Children’s Emergency Ward of the Department of Paediatrics, University of Port Harcourt Teaching Hospital (UPTH), Port Harcourt, Rivers State, Nigeria. All available medical records of poisoning cases of patients (0–14 years) who were admitted between January 2007 and June 2014 were reviewed by a researcher. The review was carried out in an allocated office within the hospital, so any ambiguity could be clarified by recording nurses. Quantitative analysis of coded data from medical records was conducted to describe pattern of poisoning and associated fiscal impact.

### Subjects

All paediatric patients admitted or attended to within the study timeline were equally liable for sampling. Inclusion criteria were patients ≤14 years of age with a main diagnosis of poisoning, whether intentional or unintentional, with or without secondary diagnosis, based on history, practitioner’s judgement or laboratory findings. All suspected poisoning cases were initially included but patients were excluded from the study if the initial diagnosis of poisoning was in doubt. Outpatient records, if available, were included. Cases of food poisoning were also included. The hospital had separate paediatric records archived in the medical records and National Health Insurance Scheme departments: these were also included. Exclusion criteria were patients >14 years of age, those with uncertain diagnosis or recorded poisoning cases without identified cause.

### Ethics

This study was approved by the University of Port Harcourt Teaching Hospital, Ethical committee, and was carried out according to standard protocol [5]. Participant anonymity was maintained throughout the study and data from the study stored in a secure location throughout study period.

### Data collection

All medical records available were reviewed for sociodemographic characteristics of patients and parents (Table 1), name, site and source of poison, time of poisoning, circumstance of exposure, time interval between poisoning and arrival at emergency room, hospital length of stay (LOS), severity, measures carried out by parents before admission, outcome and all hospital costs. These were extracted from the medical records of the hospital paediatric emergency services, medical records department and the central library. Total expenditure for each poisoning case was obtained by adding individual costs incurred: calculated as the sum of the cost of surgery, admission, pharmacy (medications), laboratory and radiology. Out-patient costs were calculated at one-third of the in-patient costs when documents were unavailable [6]. All data collected were tabulated and analysed using Microsoft excel.

**Table 1:** Family history of poisoned patients.

		N (%)

Family type	Monogamous	93
	Polygamous	3
	Single/no parent	4
Educational background	Tertiary education	52
	Secondary education	28
	Primary education	9
	No formal education	11
Employment	Employed	64
	Unemployed	36

## Results

**Figure 1:**
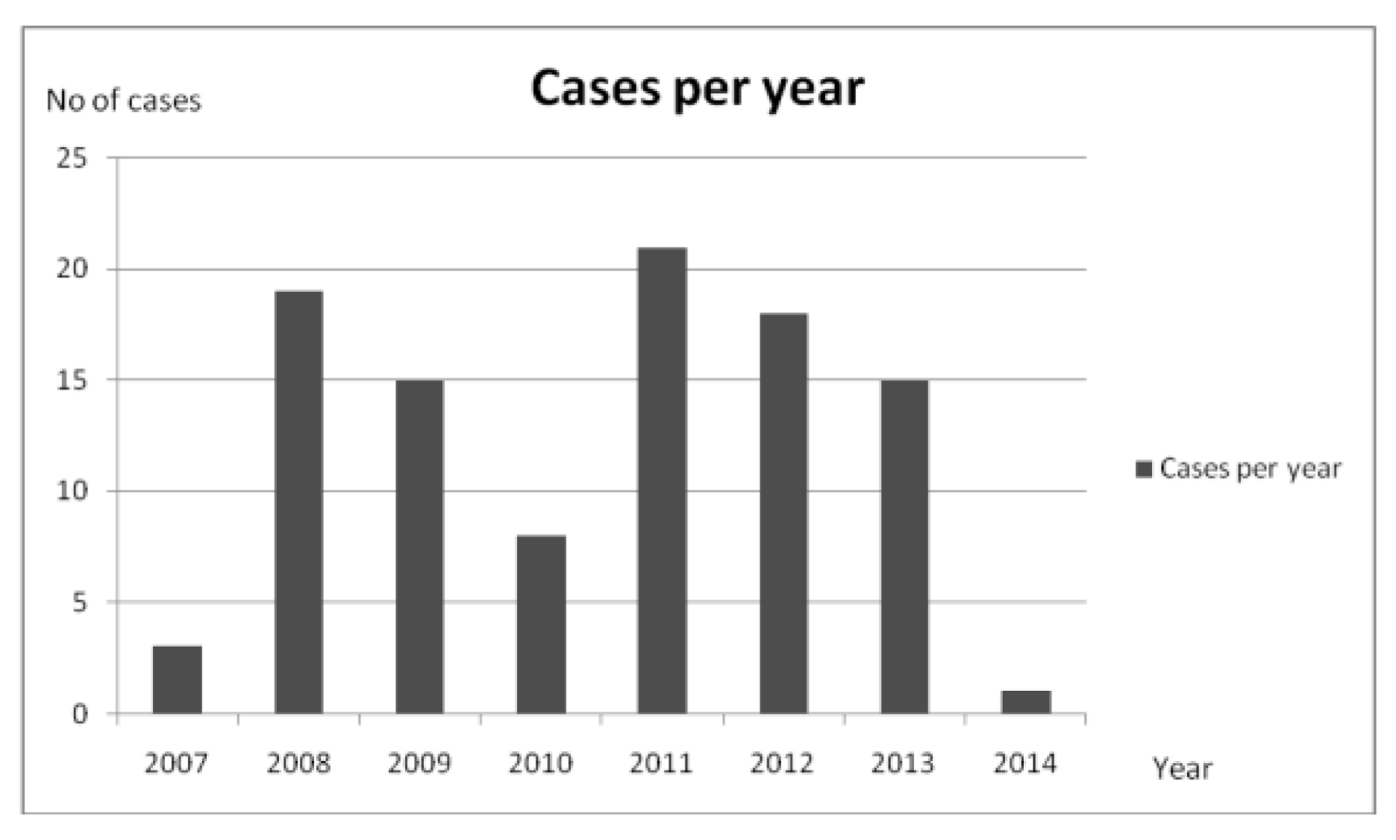
Total number of paediatric poisoning cases by year.

**Figure 2:**
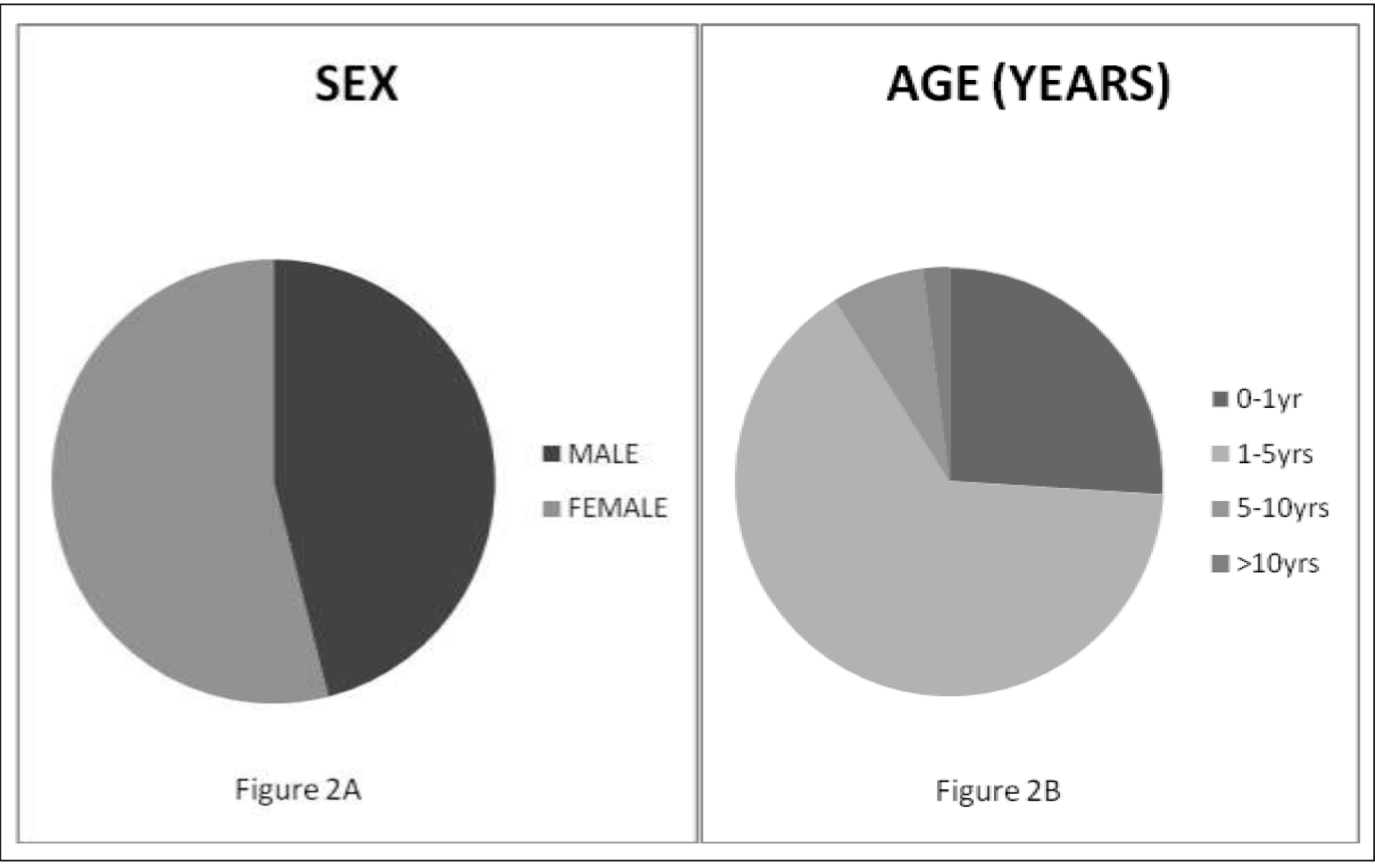
Sex and age distribution of poisoned patients.

One hundred paediatric poisoning cases were reported in UPTH, Nigeria (Figure 1) from January 2007 to June 2014. Twenty-one and eighteen cases were recorded in 2011 and 2012 respectively while three cases were recorded in 2007. Of the 100 poisoned patients, 46% were male and 54% were female, with female/male ratio of 1.17:1. The children between one and five years of age accounted for 65% of the total population (Figure 2). Ninety-three percent of the patients were from monogamous, socially stable homes; 3% were from polygamous homes; and the remaining 4% were children abandoned by one or both parents, living with relatives. Eighty-nine percent of the parents were formally educated with 52% having up to tertiary education. 64% of the parents were employed and 36% were unemployed (Table 1).

The poisoning agents (drug and non-drug), routes of exposure and clinical manifestations of poisoning are shown in Table 2. Paracetamol, amitriptyline, chlorpromazine, and ferrous sulphate were the drugs involved in poisoning. The non-drug poisons include kerosene, organophosphates, food, carbon monoxide, venom from snake bite, alcohol and rodenticides with percentage cases of 31, 13, 10, 6, 3, 2, and 2 respectively. Foreign-body inhalation accounted for 23% of the poisoning cases.

**Table 2:** Agents, routes of exposure, and clinical manifestations of poisoning.

Agent	Name	Route	Clinical manifestation	N (%)

Drug	Paracetamol	Oral	Vomiting,	74
	Amitriptyline		Hyperventilation,	
	Chlorpromazine		Seizures, Fever,	
	Ferrous sulphate		Melena, Diarrhea,	
	Others		Cough,	
Non-drug	Alcohol		Restlessness,	
	Food		Irritability,	
	Rodenticide		Unconsciousness,	
	Kerosene		Weakness	
	Foreign body	Nasal	Cough,	22
	Organophosphates		Noisy breathing,	
	Carbon monoxide		Difficulty in breathing,	
	Snake bite	Dermal	Pain, Edema, Erythema	4

About 43% of the patients were brought to the children’s emergency ward one to four hours after poisoning incident; others (33%) were referrals from private hospitals. Pre-hospital intervention measures taken by parents or care givers include giving palm oil, coconut water, milk, olive oil, activated charcoal tablet, liquid paraffin, soapy water enema to induce stooling or inducing emesis by putting a finger down the throat of the child.

At the hospital, intranasal oxygen was administered to most of the patients, gastric lavage conducted, oesophagoscopy and tracheostomy in some cases. Intravenous fluids, antibiotics, and corticosteroids were common drugs administered. Other drugs were given depending on the peculiarity of the poisoning case.

**Figure 3:**
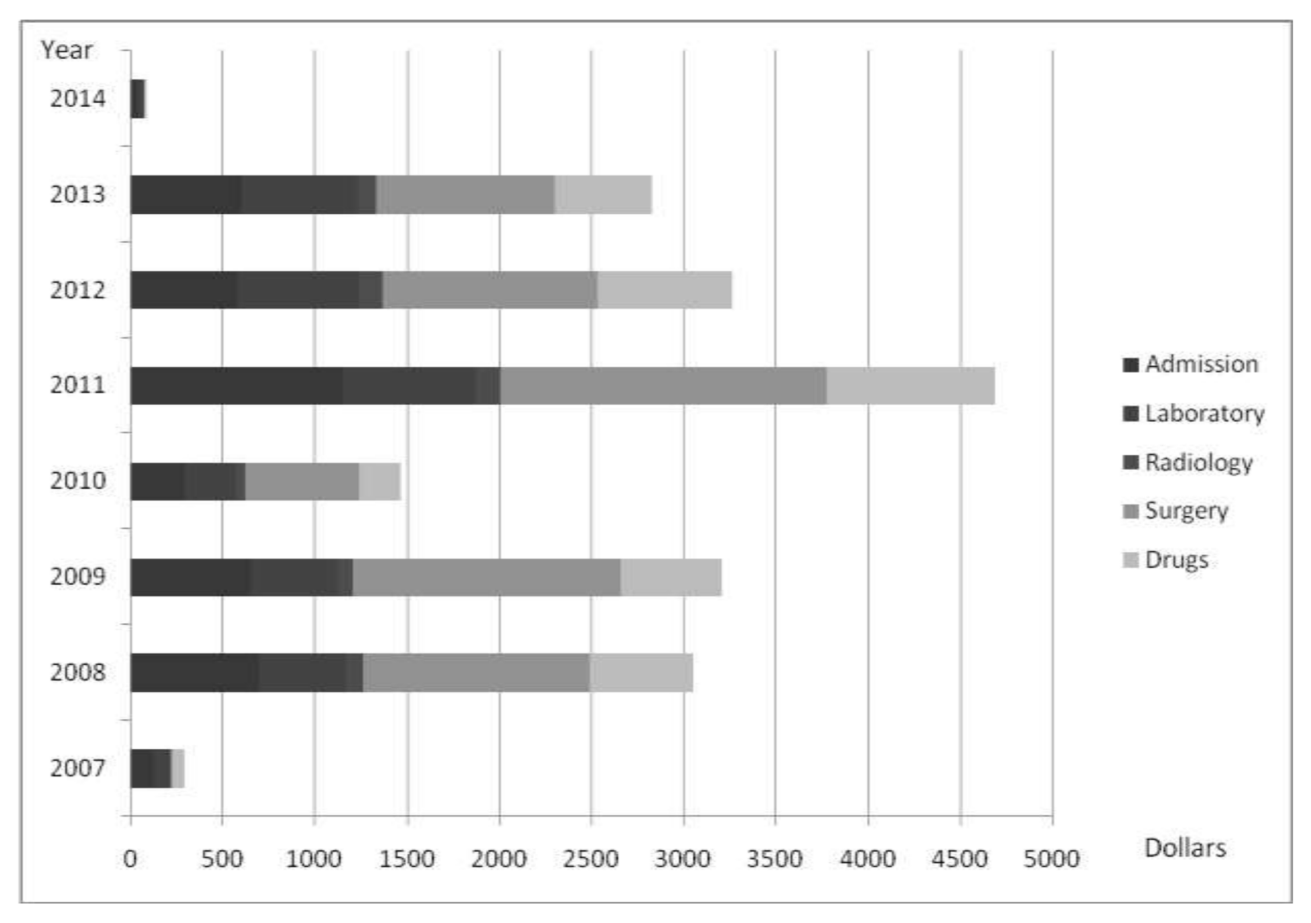
Cost of treating poisoned patients per year.

Figure 3 shows the fiscal impact of poison management per annum in University of Port Harcourt Teaching Hospital, Nigeria. About $292.70 was used to treat the poisoned patients in 2007, and $90.80 to treat the poisoned patient in 2014. The total costs for 2008, 2009, 2010, 2011, 2012 and 2013 were $3049.60, $3208.20, $1467.60, $4686.90, $3262.50, and $2828.30 respectively.

Twenty-three percent of the patients were admitted in the morning (between 12:00 a.m. and 11:59 a.m.), 17% of the cases in the afternoon (12:00 p.m. to 4:00 p.m.) and 45% in the evening/night (4:01 p.m. to 11:59 p.m.). In 15% of the cases, the time of admission was not documented. The highest percentage of patients (37%) spent less than two days on admission in the hospital and 22% of the patients spent between 2–5 days (Table 3). In 19% of the cases the hospital length of stay (LOS) was not documented, 2% of the patients absconded and 8% were discharged against medical advice. The longest duration of admission was 57 days, a case of carbon monoxide poisoning in which the patient died; the second-longest was 43 days. Thirty-seven percent of the patients were admitted for just one day and discharged. Of the 100 patients admitted, 86% recovered, 10% could not be accounted for (absconded or signed against medical advice) and 4% died.

**Table 3:** Hospital length of stay (LOS) (days) of patients.

Length of stay (los)	n (%)

<2 days	37
2–5 days	22
6–8 days	5
9–10 days	2
>10 days	5
Unknown duration	19
Signed against medical advice	8
Absconded	2
Total	100

## Discussion

This study investigated the prevalence of childhood drug and non-drug poisoning with the aim of quantifying the fiscal impact on patients. In Nigeria, as in India and South African black communities, kerosene poisoning is common and clinical spectrum can range from meager chemical pneumonitis to grave complications such as hypoxia, pneumothorax, pneumomediastinum and emphysema [7,8,9]. Kerosene was the most common toxic chemical agent implicated in the cases of childhood poisoning, similar to the study carried out by Orisakwe et al. in 2000 [8], and this could be due to its extensive use as cooking fuel and for domestic lanterns because of the high cost of cooking gas and shortage of electricity. It is also usually stored in familiar containers such as empty bottled water and soft drink containers, which may confuse children.

Drugs accounted for just 10% of the poisoning cases, which could be attributed to an increasing awareness of proper drug storage away from the reach of children. Pain killers and anti-pyretics are usually the largest group of drug toxins encountered by children [10]. Similarly, painkillers (paracetamol, ibuprofen, and aspirin) have been implicated in this study.

It was found that childhood poisoning accounted for 0.24% of all paediatric emergency cases in the University of Port Harcourt Teaching Hospital. This value is less than the 11% recorded in a research and training hospital in Istanbul, Turkey, or the 0.66%, recorded in an emergency services multicentre study in Spain [11], but greater than that reported by Gauvin et al. in Washington (0.06%) [12]. The variation in the prevalence may be explained by differences among study populations, regional differences in healthcare and quality of medical facilities [3].

The most vulnerable age group is 1–5 years (65%), which agrees with prior reports [13,14]. The natural curiosity and hand-to-mouth behaviour of this age group may explain their high vulnerability. Higher female-to-male ratio seems to be consistent with surveys of paediatric poisoning in some countries. This female predominance was also reported in Zimbabwean [15], Greek [16], Turkish [17] and Iranian [18] studies. Most hospitalizations were due to unintentional poisoning. This is similar to the high prevalence of accidental poisoning reported in both Zimbabwe [15] and Burkina Faso [14]. On the other hand, intentional poisoning was uncommon, an observation which is different from studies done in Iran and Turkey, where intentional poisoning was the most common circumstance of poisoning.

Unlike the study carried out in Patan Hospital, India, where most of the parents of the poisoned children were semi-literate artisans [19], the parents of the poisoned children in the present study were mostly educated (89%) and gainfully employed (64%), and this may account for their ability to recognise unusual behaviour in the child and bring the child to the hospital on time, as a majority of the poisoning incidents occurred when parents were away from home. The literacy level of parents in the present study may be partly responsible for their decision to consult the hospital rather than resorting to home remedy or self-care as is common with most parents in Nigeria [20].

Gauvin, et al. [12] reported a mortality rate of 0.2% among hospitalized paediatric poisoning cases. Andıran et al. [21] from Turkey reported a significant decrease in mortality in paediatric poisoning cases, from 7.6% to 0.4% over 20 years. The mortality rate of 4% in this study, which was probably due to delay in consultation of the hospital, is considered high. Food poisoning cases were all provoked by ingestion of edible foods and could be bacterial or viral in origin.

Carbon monoxide (CO) continues to be one of the most common causes of environmental poisoning throughout the world [22]. In Nigeria, where individual households provide their own electricity, the use of generators is common. These generators come in different sizes ranging from portable to very large machines often used in poorly ventilated buildings with potential for carbon monoxide poisoning. The number of cases reported in this study (six cases all from generator fumes) does not reflect the real extent of the problem. This is possibly because some die at home or en route to the hospital hence their data are not entered into hospital records. Moreover, people with asymptomatic cases may not seek consultation and unfamiliarity with CO poisoning symptoms provides frequent confusion with other diseases. These factors may have led to under reporting.

Snake and dog bites are treatable, but neglected injuries affecting predominantly tropical climes. These types of injuries are considerably more common in low and middle-income countries, largely in Asia [24]. Recent improvements in injury coding and surveillance, including community surveys in low-income and middle-income countries, have led to an increased understanding of the issue. The incidence and severity are often more with children due to their smaller body mass [23]. Analysis of 2002 WHO mortality data suggests that snake bites contribute to 35% of all child deaths, globally, with boys twice as likely to suffer as girls. Unfortunately, although the specific antidotes for snake bites, antivenoms, are organic products, there is a shortage of these products, especially in rural communities of developing countries. Antidotes of common poisoning or anti snake venom are often unavailable [3]. As a consequence, many developing countries are driven to making crude sera that are both less safe and less effective [24].

One notable feature of poison management in this study is the absence of a clearly defined standard therapeutic modality. There were no records of antidote use or use of adsorbents such as activated charcoal in the hospital despite ready availability of the latter, instead intravenous antibiotics, steroids and fluids, were given to almost all patients regardless of the cause of poisoning (drug or non-drug agents). Patients were often treated symptomatically leading to polypharmacy. Surgeries were conducted in cases of foreign-body inhalation or aspiration where there was airway obstruction or children were in obvious distress.

In most developed countries, childhood poisoning, as well as resulting treatment costs, have been extensively described. Unintentional childhood injuries in 1996 resulted in an estimated $14 billion in lifetime medical spending, $1 billion in other resource costs and $66 billion in present and future work losses in the United States. These injuries imposed quality of life losses equivalent to 92,400 child deaths [2]. Few studies have been done on the cost of poisonings, especially those affecting children or those occurring in low-income or middle-income countries. One study conducted in South Africa estimated that the direct costs alone of hospitalisation due to paraffin poisoning were at least $1.4 million per year [25]. The average cost of treatment per patient amounted to $106.50 in urban Pretoria [26] and approximately $75.58 in Cape Peninsula [27]. Data from the USA confirm the South African findings that poisonings and their management are costly [28]. The lifetime cost of poisonings in children under 15 years of age was almost $400 million with medical treatment accounting for nearly 9% of the costs. This produces a conservative estimate of $1780 on average for each case of poisoning, including medical costs, lost earnings and lost quality of life [29].

From this study, the average cost of treating a poisoned patient was $168, which is about three times the monthly minimum wage in Nigeria and higher than the cost reported in Sri Lanka ($31.83) except in the cases of pesticide poisoning ($49.12) and organophosphates ($87.44) [6]. The average admission/ward cost was $41.36, which was the highest expenditure category except in cases where surgery was conducted. This was also the highest expenditure category in Sri Lanka ($13.08) and the average cost of drugs was $31.67 in our study while it was $11.96 in Sri Lanka [6]. Diagnostics costs of $38.13, which comprised laboratory cost and radiology cost, were the second highest costs after admission cost. However, this was the least cost in Sri Lanka ($1.75) [6]. The average cost spent on all poisoned patients over the seven-year period in UPTH Port Harcourt was about $2,358.95 while in 2004, $76,599 was spent on all poisoned patients in Anuradhapura district Sri Lanka [6].

### Limitations

The inability to access complete patients’ medical records due to poor record keeping and absence of electronic records were the major challenges encountered in the present study. Only 100 recorded cases of poisoning in seven years in a tertiary hospital with huge patient patronage is considered poor. In some cases, complete clinical data could not be obtained for the poisoned children due to incomplete documentation. Truncated services occasioned by industrial actions contributed to poor record keeping. In this study, economic analysis was calculated as fiscal impact only due to the unavailability of comprehensive data and difficulty in calculating other measures in the paediatric patient demographic.

## Conclusion

Childhood poisoning in Nigeria is a costly public health concern; however, it is preventable. Prevalence and consequences can be mitigated by interventions such as proper parental education on child safety, the use of child resistant containers for the storage of toxic/poisonous substances and house hold chemicals as well as the need to keep medicines and other chemicals out of the reach of children. Establishment of conveniently located poison control centres and creating public awareness of these centres will be of immense help in checking poisoning.
